# DrugMint: a webserver for predicting and designing of drug-like molecules

**DOI:** 10.1186/1745-6150-8-28

**Published:** 2013-11-05

**Authors:** Sandeep Kumar Dhanda, Deepak Singla, Alok K Mondal, Gajendra PS Raghava

**Affiliations:** 1 Institute of Microbial Technology, Chandigarh, India; 2 Centre For Microbial Biotechnology, Panjab University, Chandigarh, India; 3 Bioinformatics Centre Institute of Microbial Technology, Sector 39A, Chandigarh, India

**Keywords:** Drug-likeness, FDA, Substructure, Fingerprints, DrugBank, SVM, Lipinski

## Abstract

**Background:**

Identification of drug-like molecules is one of the major challenges in the field of drug discovery. Existing approach like Lipinski rule of 5 (Ro5), Operea have their own limitations. Thus, there is a need to develop computational method that can predict drug-likeness of a molecule with precision. In addition, there is a need to develop algorithm for screening chemical library for their drug-like properties.

**Results:**

In this study, we have used 1347 approved and 3206 experimental drugs for developing a knowledge-based computational model for predicting drug-likeness of a molecule. We have used freely available PaDEL software for computing molecular fingerprints/descriptors of the molecules for developing prediction models. Weka software has been used for feature selection in order to identify the best fingerprints. We have developed various classification models using different types of fingerprints like Estate, PubChem, Extended, FingerPrinter, MACCS keys, GraphsOnlyFP, SubstructureFP, Substructure FPCount, Klekota-RothFP, Klekota-Roth FPCount. It was observed that the models developed using MACCS keys based fingerprints, discriminated approved and experimental drugs with higher precision. Our model based on one hundred fifty nine MACCS keys predicted drug-likeness of the molecules with 89.96% accuracy along with 0.77 MCC. Our analysis indicated that MACCS keys (ISIS keys) 112, 122, 144, and 150 were highly prevalent in the approved drugs. The screening of ZINC (drug-like) and ChEMBL databases showed that around 78.33% and 72.43% of the compounds present in these databases had drug-like potential.

**Conclusion:**

It was apparent from above study that the binary fingerprints could be used to discriminate approved and experimental drugs with high accuracy. In order to facilitate researchers working in the field of drug discovery, we have developed a webserver for predicting, designing, and screening novel drug-like molecules (http://crdd.osdd.net/oscadd/drugmint/).

**Reviewers:**

This article was reviewed by Robert Murphy, Difei Wang (nominated by Yuriy Gusev), and Ahmet Bakan (nominated by James Faeder).

## Background

High throughput screening techniques and combinatorial chemistry had provided substantial boost in our effort towards discovering new therapeutic molecules [1-3]. Despite tremendous progress in the field of drug discovery, there is a high rate of failure of drug molecules in the advanced stage of clinical trials [4,5]. Therefore, more innovative approaches are required in the process of developing new drug molecules. Among the billions of compounds that has been synthesized and tested to date, only a fraction of them has the potential to pass through the FDA approval. A recent estimate suggested that it would take more than 300 years to increase the number of available drugs by two fold at the current rate of drug discovery [6]. Therefore, a prior knowledge that could discriminate the drug-like molecules from its allies would be a welcome step for the drug discovery/design.

In the past, several attempts have been made to shrink the chemical space of the molecules having potential for drug-like properties [7]. Lipinski Rule of Five (Ro5) is the most widely accepted drug-like filter, which is based on simple analysis of four important properties of the drug molecules i.e. number of hydrogen bond donor, number of hydrogen bond acceptor, molecular weight, and solubility [8]. Although, Ro5 had been used as a major guideline in the drug discovery efforts, it has also several limitations [9]. This method is not universally applicable and many compounds particularly those from natural origin e.g. antibiotics etc. are not recognized by this method as drug-like compounds [10]. Recently, it has also been reported that among the two hundred best selling branded drugs in 2008, twenty one had violated Ro5 [11]. Previously, it has been reported that the real drugs are ~20-fold more soluble than the drug-like molecules present in the ZINC database. Specifically, the oral drugs are about 16-fold more soluble, while the injectable drugs are 50–60 fold more soluble [12]. Comparison of the two molecular properties i.e. molecular weight and ClogP, for different families of FDA-approved drugs, suggested that the modified rules of drug-likeness should be adopted for certain target classes [13]. In 2008, Vistoli *et al.* summarized the various kindS of pharmacokinetic and pharmaceutical properties of the molecules playing an important role in estimation of drug-likeness [14]. Recently, Bickerton *et al.* developed a simple computational approach for prediction of oral drug-likeness of the unknown molecules [11]. This is very simple approach applicable only for the oral drugs.

In order to overcome these problems, several models based on machine learning techniques have been developed in the past. An earlier computational model developed in 1998 for predicting drug-like compounds was based on simple 1D/2D descriptors, which showed a maximum accuracy of 80% [15]. In the same year, another study also tried to predict the drug-like molecules based on some common structures that were absent in the non-drug molecules [16]. Genetic algorithm, decision tree, and neural network based approaches had also been attempted to distinguish the drug-like compounds from the non drug-like compounds [17-19]. These approaches, although used a large dataset, only showed a maximum accuracy up to 83%. In comparison, better success was shown by some recent studies in predicting drug-like molecules. In 2009, Mishra *et al.* had classified drug-like small molecules from ZINC Database based on “Molinspiration MiTools” descriptors using a neural network approach [20]. The other reports that appeared promising in predicting the potential of a compound to be approved were based on DrugBank data [21,22].

The main problem associated with the existing models is their non-availability to the scientific community. Moreover, the commercial software packages were used to develop these models, so these studies have limited use for scientific community. In order to address these problems and to complement previous methods, we have made a systematic attempt to develop a prediction model. The performance of our models is comparable or better than the existing methods.

## Results and discussion

### Analysis of dataset

#### Principal Component Analysis (PCA)

**Figure 1:**
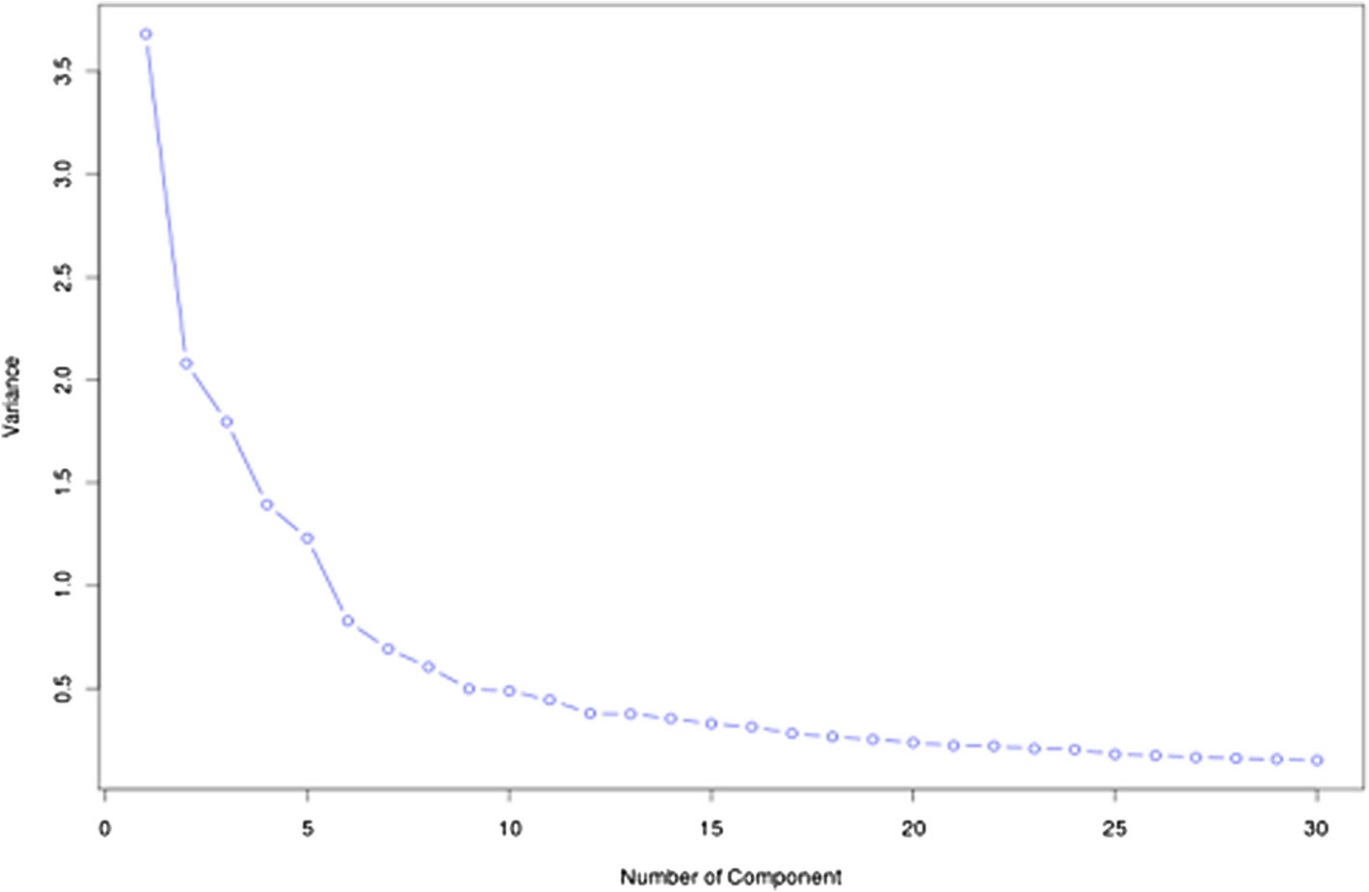
Variance of components in our dataset.

**Figure 2:**
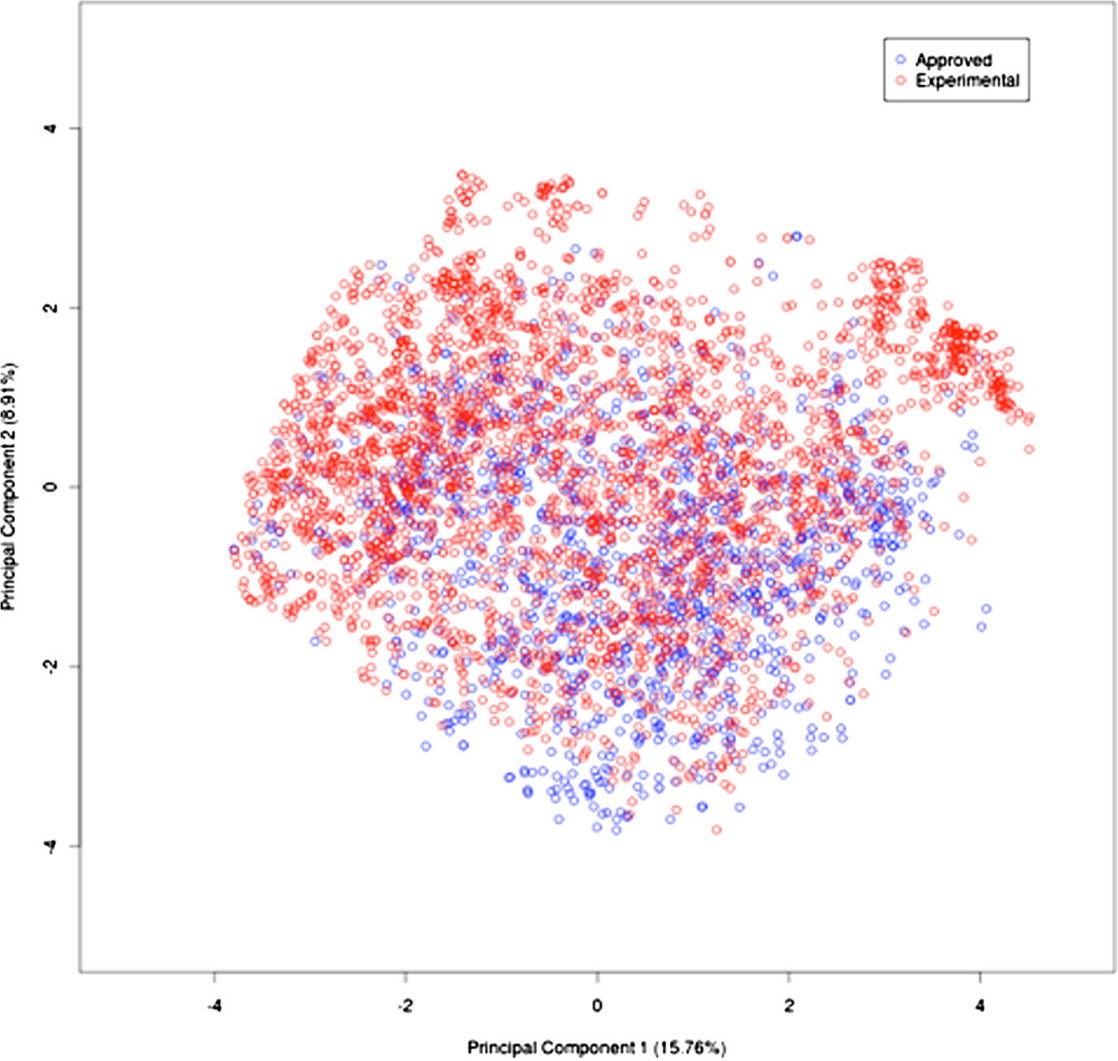
Two-dimensional plot of Principal Component Analysis for approved and experimental drugs, each drug molecule is represented by circle.

We used the principal component analysis (PCA) for computing the variance among the experimental and the approved drugs [23]. As shown in Figure 1, the variance decreased significantly up to the PC-15. Afterwards, it remained more or less constant. The variance between PC-1 and PC-2 for the whole dataset was 15.76% and 8.91% respectively [Figure 2]. These results clearly indicated that the dataset was highly diverse for developing a prediction model.

#### Substructure fragment analysis

To explore the hidden information, the dataset was further analyzed using SubFP, MACCS keys based fingerprints using the formula given below;

$$\mathrm{Frequency}\;\mathrm{of}\;a\;\mathrm{fragment}=\left(N_{\mathrm{fragment}\_\mathrm{class}}*N_{\mathrm{total}}/N_{\mathrm{fragment}\_\mathrm{total}}*N_{\mathrm{class}}\right)$$

Where N_fragment_class_ is the number of fragments present in that class (approved/experimental); N_total_ is the total number of molecules studied (approved + experimental); N_fragment_total_ is the total number of fragments in all molecules (approved + experimental); N_class_ is the number of molecules in that class (approved/experimental).

**Figure 3:**
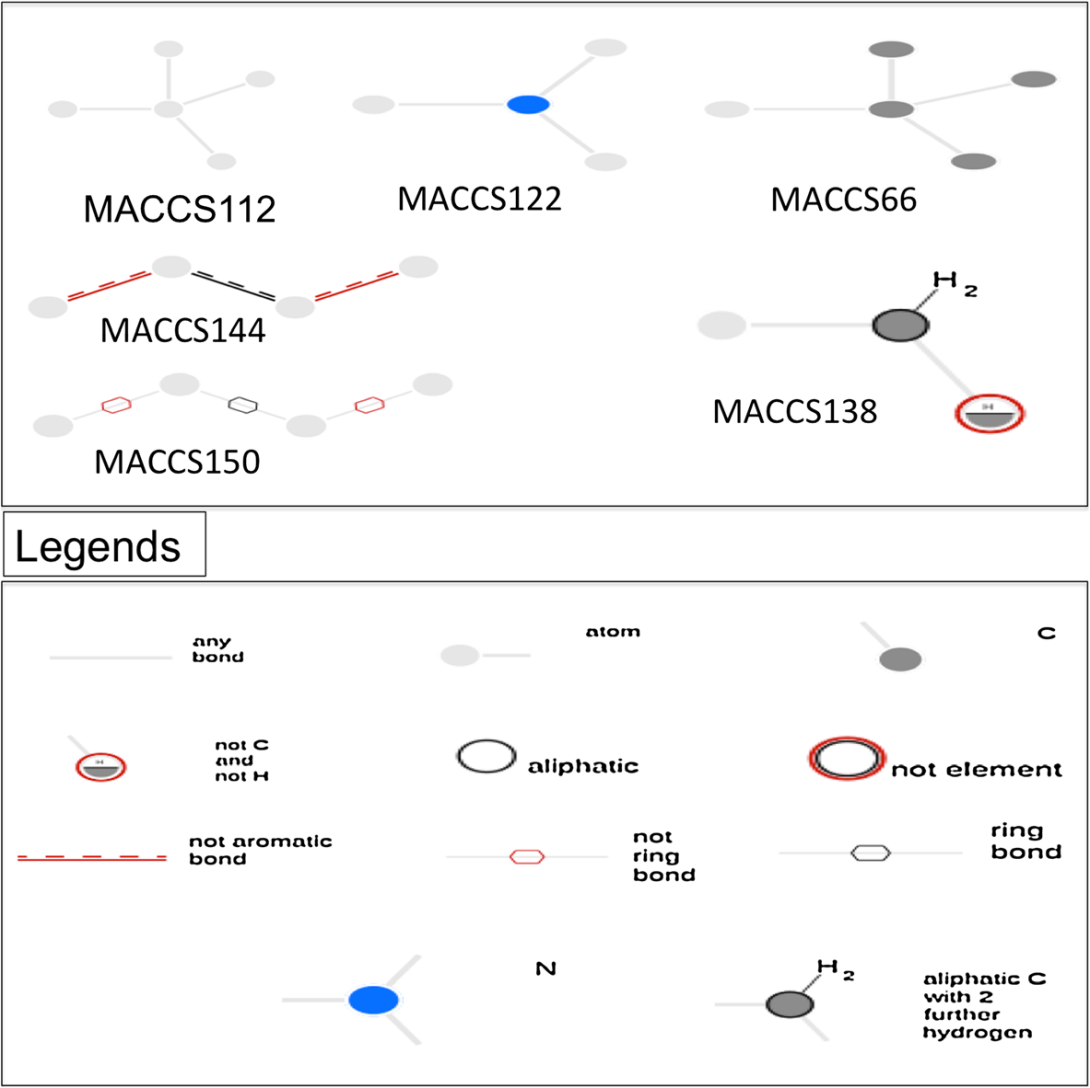
Representation of the selected MACCS keys.

Our analysis suggested that some of the substructure fragments were not preferred in the approved drugs. The substructure-based analysis suggested that primary alcohol, phosphoric monoester, diester and mixed anhydride were non-preferable functional groups that were present in the experimental drugs with higher frequency [Table 1]. Similarly, MACCS keys 66, 112, 122, 138, 144, and 150 were highly desirable and present with higher frequency in the approved drugs [Table 2, Additional file 1: Table-S1 and Figure 3]. Therefore, while designing new drug-like molecule in the future, the exclusion of SubFP fingerprints and the inclusion of certain MACCS keys might increase the probability of designing a better molecule.

**Table 1:** **Top-10 Substructure fingerprints and their respective frequency in our dataset**^**a**^

**SubFP**	**Description**	**Approved**		**Experimental**		**Frequency difference**
		**N**_**frag_aprd**_	**F**_**aprd**_	**N**_**frag_exp**_	**F**_**exp**_	**(F**_**aprd **_**-F**_**exp**_**)**
**SubFP13**	Primary_alcohol	139	0.69	539	1.13	-(0.44)
**SubFP41**	1-2Diol	67	0.31	663	1.29	-(0.98)
**SubFP48**	Aldehyde	4	0.16	79	1.35	-(1.19)
**SubFP126**	Alpha_hydorxyacid	1	0.07	50	1.39	-(1.32)
**SubFP224**	Sulfenic_derivatives	23	0.59	108	1.17	-(0.58)
**SubFP237**	Phosphoric_monoester	5	0.06	289	1.4	-(1.34)
**SubFP238**	Phosphoric_diester	3	0.2	47	1.33	-(1.13)
**SubFP246**	Phosphoric_acid_derivatives	16	0.1	506	1.38	-(1.28)
**SubFP281**	Sugar_pattern_1	71	0.34	627	1.28	-(0.94)
**SubFP291**	Mixed_anhydride	3	0.05	214	1.4	-(1.35)

^a^N_frag_aprd_: number of fragment in approved class, N_frag_exp_: number of fragment in experimental class, F_aprd_: frequency of particular fragment in approved class, F_exp_: frequency of particular fragment in experimental class, negative sign indicates that these fingerprints are not preferable in approved drug.

**Table 2:** **Highly significant MACCS fingerprints and their respective frequency in our dataset**^**a**^

**Fingerprint**	**Accuracy (%)**	**Approved**		**Experimental**		**Frequency difference**
		**N**_**frag_aprd**_	**F**_**aprd**_	**N**_**frag_exp**_	**F**_**exp**_	**(F**_**aprd **_**- F**_**exp**_**)**
MACCS112	66.35	1288	1.58	1473	0.76	0.82
MACCS122	66.66	1105	1.57	1276	0.76	0.81
MACCS144	69.21	972	1.64	1027	0.73	0.91
MACCS66	73.97	557	1.98	395	0.59	1.39
MACCS150	57.57	1227	1.36	1812	0.85	0.52
MACCS138	65.91	910	1.52	1115	0.78	0.74

^a^N_frag_aprd_: number of fragment in approved class, N_frag_exp_: number of fragment in experimental class, F_aprd_: frequency of particular fragment in approved class, F_exp_: frequency of particular fragment in experimental class.

### Classification models

In order to evaluate the performance of different fingerprints, we have developed various models on different sets of descriptors that were calculated by PaDEL software. Separate models were developed on fingerprints selected using attribute selection modules rm-useless and CfsSubsetEval of Weka.

### Fingerprints based models

**Figure 4:**
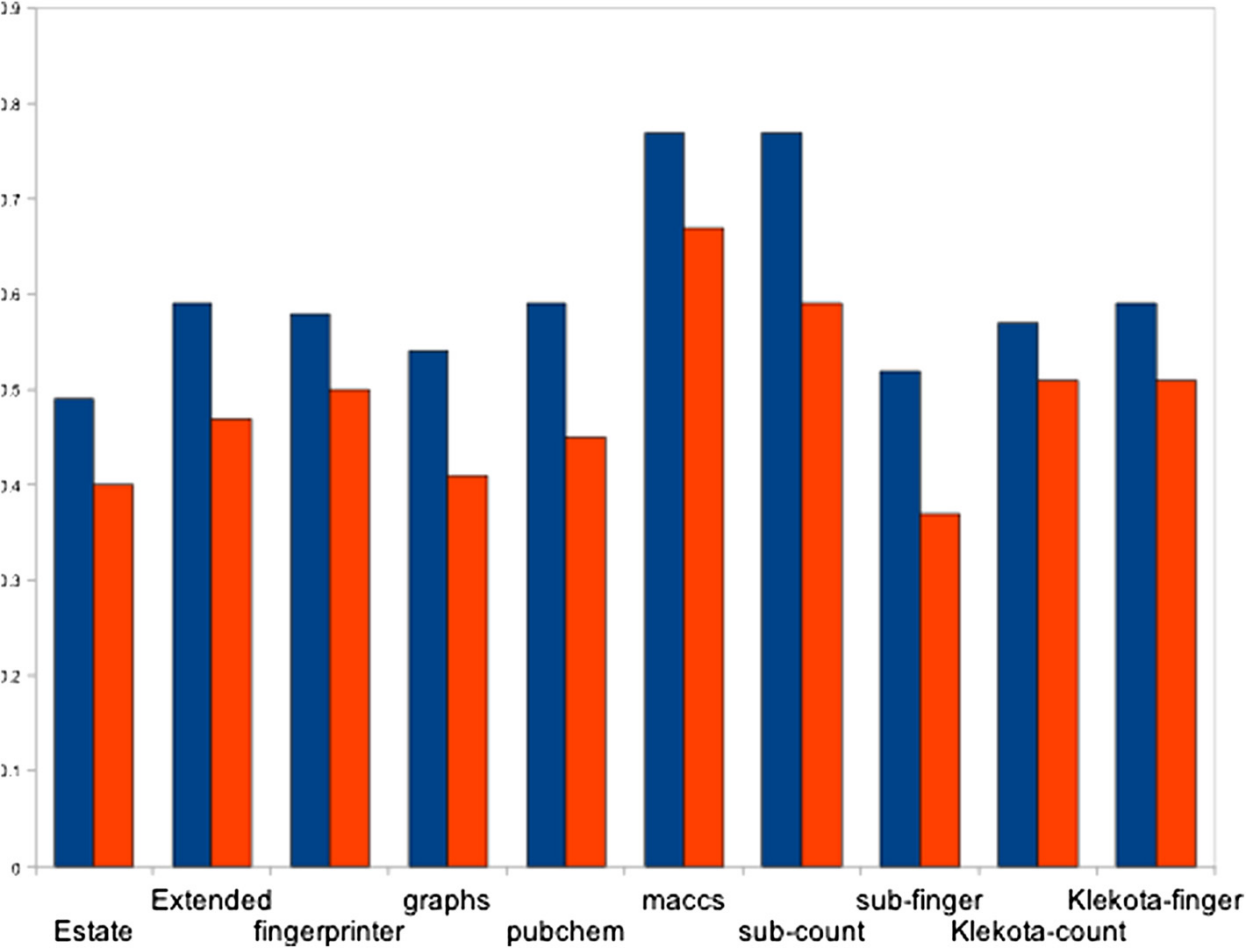
Various sets of descriptors versus Matthew’s Correlation Coefficient (MCC).

The initially developed models based on Estate, PubChem, Extended, FingerPrinter, GraphsOnly, Substructure finger, Substructure count, Klekota-count, Klekota-fingerprint showed nearly equal performance with MCC value in the range of 0.5 to 0.6 [Table 3]. However, the models developed using 159 MACCS keys, achieve maximum MCC 0.77 with accuracy 89.96% [Table 3, Figure 4]. In addition to that, we have also applied Monte-Carlo (MC) approach by generating 30 times training and testing dataset for five-fold cross-validation. We have observed that these results were more or less same with previously used five-fold cross-validation results having average 87.88%/90.36% sensitivity/ specificity, 89.63% accuracy with MCC value 0.76 (Additional file 1: Table-S2).

**Table 3:** Performance of various Fingerprints and selection-algorithm

**Fingerprints**	**Selection-algorithm**	**Descriptors**	**Threshold**	**Sensitivity**	**Specificity**	**Accuracy**	**MCC**	**AUC**
**Estate**	rm-useless	52	0	70.23	80.6	77.53	0.49	0.82
	cfsSubsetEval	9	0.3	70.9	71.68	71.45	0.4	0.77
**Extended**	rm-useless	1012	0	62.44	92.51	83.62	0.59	0.86
	cfsSubsetEval	25	−0.4	60.13	85.93	78.3	0.47	0.79
**Fingerprinter**	rm-useless	1024	0	61.99	92.36	83.37	0.58	0.86
	cfsSubsetEval	40	−0.2	63.62	85.84	79.27	0.5	0.81
**GraphsOnly**	rm-useless	1024	0	67.78	86.28	80.8	0.54	0.85
	cfsSubsetEval	43	0	69.86	73.71	72.57	0.41	0.78
**Pubchem**	rm-useless	704	0	63.85	92.11	83.75	0.59	0.87
	cfsSubsetEval	27	0.4	66.59	79.76	75.86	0.45	0.8
**MACCS**	rm-useless	159	0	88.42	90.61	89.96	0.77	0.95
	cfsSubsetEval	10	0	89.83	81.72	84.12	0.67	0.87
**Substr-count**	rm-useless	192	0	93.1	87.84	89.39	0.77	0.95
	cfsSubsetEval	16	−0.3	84.71	78.32	80.21	0.59	0.88
**Sub-finger**	rm-useless	192	0	76.32	78.45	77.82	0.52	0.84
	cfsSubsetEval	18	0	50.71	84.44	74.46	0.37	0.74
**Klekota-count**	rm-useless	2273	−0.2	63.92	90.42	82.58	0.57	0.85
	cfsSubsetEval	57	0	72.31	80.82	78.3	0.51	0.82
**Klekota-finger**	rm-useless	2273	0	61.84	92.89	83.7	0.59	0.86
	cfsSubsetEval	51	0	53.75	91.98	80.67	0.51	0.81

### PCA based model

In the previous section, we have observed that the models developed using MACCS keys based fingerprints perform better in comparison to the models developed using other fingerprints. We used this class of fingerprint for developing a PCA based model. First model, which was developed on all 166 components, achieved maximum MCC 0.79 and ROC 0.96 [Table 4]. The models developed using top-20 fingerprints [Figure 1], achieved maximum MCC 0.72 with a marginal decrease in the value of ROC to 0.94. Furthermore, the models developed using top-15, and top-10 components resulted in a MCC value of 0.68 and 0.61 respectively. A slight decrease in the MCC value was observed on further reducing the number of components to 5.

**Table 4:** Performance of PCA based models on MACCS descriptors

**Selection-algorithm**	**Descriptors**	**Threshold**	**Sensitivity**	**Specificity**	**Accuracy**	**MCC**	**AUC**
**ALL**	166	−0.1	91.24	90.33	90.60	0.79	0.96
**Top-20**	20	0.0	82.93	90.05	87.94	0.72	0.94
**Top-15**	15	0.0	85.97	85.03	85.31	0.68	0.92
**Top-10**	10	0.0	86.27	78.95	81.11	0.61	0.88
**Top-5**	5	−0.1	75.95	80.57	79.20	0.54	0.84

### Hybrid models

In this section, we described hybrid models developed by combining the descriptors that were selected from Table 3. First, a Hybrid model (Hybrid-1) was developed using the top-5 positively correlated fingerprints from each (10 types of) class and this model obtained MCC up to 0.7. Second hybrid model (Hybrid-2) based on the top-5 negatively correlated descriptors achieved MCC value 0.36 [Table 5]. A third hybrid model (Hybrid-3) was developed by combining the top-5 positively and the top-5 negatively fingerprints and it resulted in a slight increase in the performance in comparison to the individual ones and showed a MCC value of 0.77 [Table 5].

**Table 5:** **Performance of various hybrid models developed using combination of descriptors**^**b**^**.**

**Model**	**Descriptors**	**Threshold**	**Sensitivity**	**Specificity**	**Accuracy**	**MCC**	**AUC**
Hybrid-1	50	0	86.86	86.28	86.45	0.7	0.92
Hybrid-2	50	0	74.09	65	67.69	0.36	0.73
Hybrid-3	100	−0.1	92.43	87.99	89.3	0.77	0.95
Hybrid-4	296	0	90.57	89.86	90.07	0.78	0.96
Hybrid-5	22	0	87.75	84.68	85.59	0.69	0.9

^b^Hybrid-1: top 5 descriptors from each fingerprints based on their positive correlation against activity, Hybrid-2: top 5 descriptors from each fingerprints based on their negative correlation against activity, Hybrid-3: sum of descriptors from Hybrid-1 and Hybrid-2, Hybrid-4: sum of all 10 types of fingerprints after applying CfsSubsetEval algorithm, Hybrid-5: Running the CfsSubsetEval algorithm on the descriptors set of Hybrid-4 (296).

Next, by combining the descriptors of CfsSubsetEval module for each fingerprint, a hybrid model (Hybrid-4) was developed which showed accuracy up to 90.07% with a MCC value of 0.78 [Table 5]. Finally, a hybrid (Hybrid-5) model on 22 descriptors was obtained upon further reducing these descriptors (296) by CfsSubsetEval module and it resulted in a slight decrease in MCC value to 0.7 with a significant reduction in the number of descriptors.

### Performance on validation dataset

We evaluated the performance of our three; i) rm-useless, ii) PCA based, and iii) CfsSubsetEval based models using validation dataset created from MACCS fingerprints (see detail in material and method section). Each model were trained and validated by internal five-fold cross validation [Table 6]. The best-selected models were further used to estimate the performance on validation dataset. The first model based on 159 (rm-useless) fingerprints showed sensitivity/specificity 90.37%/87.21% with MCC value 0.77 on validation dataset. Next, model was built on top 20 PCs shows sensitivity/specificity 81.85%/87.21% with MCC value 0.67 [Table 6]. However, the CfsSubsetEval based model developed on 10 fingerprints shows maximum MCC 0.62 on validation dataset. This decrease in MCC value on validation dataset might be due to reduction in number of descriptors.

**Table 6:** Performance of Models on New training and validation dataset built using MACCS fingerprints

**Dataset**	**Sensitivity**	**Specificity**	**Accuracy**	**MCC**	**AUC**
**Model based on 159 MACCS keys after rm-useless**					
New train	90.25	89.40	89.65	0.77	0.95
Validation	90.37	87.21	88.14	0.77	0.95
**Model based on Top-20 PCs**					
New train	85.70	88.65	87.78	0.72	0.94
Validation	81.85	87.21	85.62	0.67	0.92
**Model based on 10 MACCS keys after CfsSubsetEval**					
New train	89.42	81.83	84.07	0.67	0.89
Validation	84.07	81.44	82.22	0.62	0.88

### Performance on independent dataset

We tested our MACCS (ISIS) keys based model on the independent dataset and achieved 84% sensitivity, 38.92% specificity with accuracy value of 41.15%. These results also indicated that ~61% of the molecules present in our independent dataset have the potential to be in the approved category in future. Recently, twenty-one drugs were approved in the DrugBank v3.0, which was not classified as approved in the earlier release. Interestingly, all these compounds were classified in the ‘drug-like’ class by our model and this result clearly exemplified the performance of our model. Together, these results also indicated that our model could be very useful in the prediction of drug-like properties of a given compound in advance.

### Screening of databases

We predicted drug-like potential of molecules in three major databases ChEMBL, ZINC and directory of useful decoys (DUD). The screening of 10384763 compounds from ZINC database showed that 78.33% (8134753 molecules) among them have the potential to be drug-like (http://crdd.osdd.net/oscadd/drugmint/data/zinc.csv). Similarly, ChEMBL dataset contained 1251913 molecules, only 72.43% (906791 molecules) were predicted to have drug-like properties (http://crdd.osdd.net/oscadd/drugmint/data/chembl.csv). Finally, our software predicted ~62% and ~64% of the compounds that are present in active and decoys datasets respectively to be drug-like [Additional file 2]. These results indicated that despite the growth of a large number of chemicals showing pharmacological activity in a particular condition, not all molecules have potential for satisfying the drug-like properties.

## Conclusions

This study showed that a better predictive model for discriminating the approved drug from the experimental drugs could be developed using simple binary fingerprints. In terms of sensitivity, specificity, accuracy as well as MCC values, the performance of our model was better than those described earlier in the literature. Moreover, this could be achieved with ~50% reduction in the number of descriptors which is highly significant. Our study also suggested that the CfsSubsetEval algorithm could be used for the selection of the informative descriptors to increase the speed of calculation without compromising the efficiency of the model. From the PCA based models, we observed that 20 PCs were sufficient to develop a prediction model. We have also evaluated the performance of QED method on datasets used in this study, QED correctly classified 44.8% approved and 81.28% experimental drugs from the training dataset and 40% approved and 52.5% experimental drugs from the independent dataset. The performance of QED particularly sensitivity was very poor, it might be due to that QED approach was specifically developed for oral drugs whereas our datasets contained all types of drugs. Among the various numbers of selected fingerprints, some were preferable in the approved drugs while others on the experimental drugs. In addition to that our MACCS keys based model correctly predicted the twenty-one drugs recently listed by FDA in the approved category. Similarly on the independent dataset, our model performed with sensitivity values up to 84%. Our analysis suggested that primary alcohol, phosphoric monoester, diester and mixed anhydride were non-preferable functional groups. The efficiency of the freely available software was quite similar to that of the commercially available software. We predict that this webserver will be useful in future for selecting the drug-like molecules.

### Web server

The major drawback of most of chemo-informatics studies is that they are mainly based on commercial software packages. This is the reason most of the predictive studies described in literature are not available for public use in the form of software or web server. In order to overcome this drawback, we have used freely available software and achieved results comparable to those that have used commercial software. Our study is implemented in the form of a webserver without any restriction. In this server, we have provided the facility to design, screen and predict the drug-likeness score of chemical compounds. The screening results of ZINC and ChEMBL library are also provided in the option of database search. In order to provide this free service to the community, we have developed “drugmint” (http://crdd.osdd.net/oscadd/drugmint) a user-friendly webserver for discriminating the approved drug from the experimental drugs. This server allows users to interactively draw/modify a molecule using a Marvin applet [24]. This server is installed on Linux (Red Hat) operating system. The common gateway interface (CGI) scripts of “drugmint” are written using PERL version 5.03.

## Methods

### Dataset source

#### Training dataset

The dataset used in this study was taken from *Tang et al.*[22], contained 1348 approved and 3206 experimental drugs derived from DrugBank v2.5. The PaDEL software was unable to calculate the descriptors of one approved drug with DrugBank ID DB06149. Therefore, we did not include this molecule in our final dataset, comprises of 1347 approved and 3206 experimental drugs.

#### Validation dataset

We have also created a validation dataset from the final dataset by randomly taking 20% of data from the whole dataset. Thus, our new training dataset consist of 1077 approved, 2565 experimental drugs and validation dataset comprises of 270 approved and 641 experimental drugs.

#### Independent dataset

We also created an independent dataset from DrugBank v3.0. Initially, all the 1424 approved and 5040 experimental drugs from DrugBank v3.0 were extracted. All molecules used in our main or training dataset were removed and finally we got 237 approved and 1963 experimental drugs. Our final independent dataset comprises of 100 approved and 1925 experimental drugs after excluding the compounds for which structure was not available in the database.

### Descriptors of molecules

In this study, PaDEL was used for calculating the descriptors of the molecules [25]. This software computed approximately 800 descriptors (1D/2D/3D) and 10 types of fingerprints (e.g., Fingerprinter, Extended, GraphOnly, SubStructure, Substructure count, PubChem, MACCS keys [26], KlekotaRoth , KlekotaRoth count, Estate). The number of descriptors in each type of fingerprint is given in Table 7.

**Table 7:** Shows the number of descriptors present in each type of fingerprint

**Fingerprint name**	**Fingerprints count**	**Fingerprint name**	**Fingerprints count**
**Fingerprinter**	1024	**PubChem**	881
**Extended fingerprinter**	1024	**MACCS**	166
**GraphOnly**	1024	**KlekotaRoth FP**	4860
**SubStructure FP**	307	**KlekotaRoth FPcount**	4860
**Substructure fingerprintercount**	307	**Estate**	79

### Selection of descriptors

It has been shown in previous studies that all descriptors are not relevant [27]. Thus, the selection of descriptors is a crucial step for developing any kind of prediction model [28,29]. In this study, we used two modules of Weka i) Remove Useless (rm-useless) and ii) CfsSubsetEval with best-fit algorithm [30]. In case of rm-useless, all those descriptors, which either varies too much or variation is negligible, have been removed. The CfsSsubsetEval module of Weka is a rigorous algorithm; it selects only those features or descriptors that have high correlation with class/activity and very less inter-correlation.

### Cross-validation techniques

Leave one out cross-validation (LOOCV) is a preferred technique to evaluate the performance of a model. This technique is time consuming and CPU intensive particularly when dataset is large. In this study, we have used five-fold cross-validation technique to reduce the computational time for developing and evaluating our models. In this technique, the whole data set is randomly divided into five sets of similar size, four sets are used for training and remaining set for testing. This process is repeated five times in such a way that each set is used only once for testing. Overall performance is computed on the whole dataset after repeating the aforesaid process five times.

### Model development

In this study, we have developed Support Vector Machine (SVM) based models for prediction of drug-like molecules using SVM^light^ software package. SVM is based on the statistical and optimization theory and it handles complex structural features, and allows users to choose a number of parameters and kernels (*e.g.* linear, polynomial, radial basis function, and sigmoid) or any user-defined kernel. This software can be downloaded freely from http://www.cs.cornell.edu/People/tj/svm_light/.

### Evaluation parameters

All the models developed in this study were evaluated using standard parameters such as i) Sensitivity (percentage of correctly predicted approved drug), ii) Specificity (Percentage of correctly predicted experimental drug), iii) Accuracy (percentage of correctly predicted drugs) and iv) Matthew’s Correlation Coefficient (MCC). These parameters can be calculated using following equations 1 to 4.

(1) $$\mathrm{Sensitivity}=\frac{\mathrm{TP}}{\mathrm{TP}+\mathrm{FN}}\times 100$$

(2) $$\mathrm{Specificity}=\frac{\mathrm{TN}}{\mathrm{TN}+\mathrm{FP}}\times 100$$

(3) $$\mathrm{Accuracy}=\frac{\mathrm{TP}+\mathrm{TN}}{\mathrm{TP}+\mathrm{TN}+\mathrm{FP}+\mathrm{FN}}\times 100$$

(4) $$\mathrm{MCC}=\frac{\left(\mathrm{TP}\times \mathrm{TN}\right)-\left(\mathrm{FP}\times \mathrm{FN}\right)}{\sqrt{\left[\left(\mathrm{TP}+\mathrm{FN}\right)\left(\mathrm{TN}+\mathrm{FP}\right)\left(\mathrm{TP}+\mathrm{FP}\right)\left(\mathrm{TN}+\mathrm{FN}\right)\right]}}$$

where TP and TN are the number of truly or correctly predicted positive (approved) and negative (experimental) drugs, respectively. FP and FN are the number of false or wrongly predicted approved and experimental drugs, respectively. Matthew’s correlation coefficient (MCC) is considered to be the most robust parameter of any class prediction method. We have also used a threshold-independent parameter called receiver-operating curve (ROC) for evaluating performance of our models.

## Reviewers’ comments

### Reviewer number 1: Dr Robert Murphy

Comment-1: This manuscript describe a fairly simply design of a machine learning system for predicting whether a chemical structure is similar to previously approved drugs. It describes a web server to provide predictions about new structures.

The manuscript does not provide sufficient discussion of relevant prior work and quantitative comparison with other published approaches for which code is available (e.g., Bickerton *et al.* 2012). Approaches such as features reflecting drug dynamics (e.g., Vistoli *et al.* (2008) Drug Discovery Today 13:285–294 (doi:10.1016/j.drudis.2007.11.007) are also not discussed.

Response: In the revised version, we have discussed the previous studies as suggested by reviewer. After getting comments from the reviewer, we evaluate performance of QED model on our datasets, QED correctly predict 44.8% (sensitivity) approved and 81.28% (specificity) experimental drugs. While on independent dataset, it shows only 40% sensitivity and 52.5% specificity. QED (Bickerton *et al. 2012*) perform poor on our dataset because it is developed for predicting oral drug-likeness of a molecule. The high sensitivity and specificity of our models described in this study implies its usefulness in predicting drug-likeness of a molecule.

Comment-2: There is a potentially serious concern with the validity of the results due to the fact that the experimental design may result in overfitting. Even though cross-validation was used internally for combinations of features and learners to evaluate predictive accuracies, when these results are subsequently used to make decisions (such as which features to use) it compromises any conclusions from further analysis of the same training and testing data. A related problem may also arise from maximization of ROC area when some of the experimental drugs may indeed be drug-like. These concerns were shown to be warranted because the final evaluation using an independent dataset showed much lower accuracy. However, it is somewhat encouraging that twenty-one molecules in the test set that were recently approved as drugs were classified as “drug-like” by the authors’ model.

Response: We are thankful to reviewer for this valuable comment. In order to further validate our prediction model, we used Monte-Carlo approach where we randomly create training and testing datasets 30 times and compute average performance. We achieved sensitivity 87.88%, specificity 90.36% and accuracy 89.63% when evaluated using Monte-Carlo approach. The result for every set is provided in supplementary document (Additional file 1: Table-S2) in the form of sensitivity, specificity, accuracy and MCC along with their mean and standard deviation. These results were more or less same to the previous five fold results. The result indicates that our models are not over-fitted and will be useful in real scenario.

Comment-3: The web server model does not seem appropriate for the primary use case, which is envisaged to be making predictions for users with novel structures. Since users may wish to keep their structures private, an open source approach would be strongly preferable to a public server. This would secure use of the system and also permit inspection and modification of the methods used.

Response: We are thankful for this suggestion. We understand the limitation of the webserver used for prediction. In order to facilitate and for the sake of user privacy, we developed a standalone version of this software, which is available for download from http://osddlinux.osdd.net, now user can run our software on their local machine.

Additional comment-1: The author list contains “Open Source Drug Discovery Consortium” which is not a person and is not mentioned elsewhere in the manuscript.

Response: We are thankful for this comment. In the revised version, we have acknowledged the Open Source Drug Discovery Consortium instead of authors list.

Additional comment-2: The abstract refers to screening but the manuscript does not describe any screening results.

Response: The authors are thankful for this suggestion. In the revised manuscript, we have provided the detailed of chemical libraries and their screening results under the paragraph screening of databases.

Quality of written English: Needs some language corrections before being published.

Response: We have corrected the language in the revised manuscript.

### Reviewer number 2: Prof Difei Wang (nominated by Dr Yuriy Gusev)

In general, this is an interesting work and it is important to predict drug-like molecules using various types of molecular fingerprints. However, I do have some questions about the manuscript.

Comment-1: On page7, the authors stated that “Similarly, MACCS fingerprint elements 112, 122, 144, and 150 were highly desirable and present with higher frequency in the approved drugs [Table 2, Figure 3]”. How to interpret this observation? What are the definition of MACCS-144 and −150 etc.? It will be very useful if the authors can clearly explain what are these features. Also, MACCS-66 is missing here but it does show up in the Table. Is there any reason to exclude MACCS-66 here?

Response: We are thankful to the reviewer for this nice suggestion. Here, we are providing the selected MACCS keys description that would be useful to interpret the results [Additional file 1: Table-S1].

a) MACCS 66: A tetrahedral carbon atom connected with 3 carbons and one (that may or may not be carbon) atom.

b) MACCS 112: Any atom connected with four atoms by any kind of bond (single, double or triple).

c) MACCS 122: A nitrogen atom joined with 3 other atoms by any kind of bond.

d) MACCS 138: An aliphatic carbon connected with 3 atoms of which one atom is not the carbon or hydrogen, second is any atom and third is with 2 further hydrogen’s.

e) MACCS 144: Any four atoms connected by non-aromatic bonds.

f) MACCS 150: Any four atoms connected of which atom 1,2 and 3,4 connected by non-ring bond and atom 2,3 joined by ring bond.

Comment-2: What is the score cutoff value for drug like and non drug like molecules for database screening results? What are the meaning of “drug like, low”, “drug like, high” and “non drug like, low”? What false-positive rate do we expect here?

Response: The authors are thankful for this comment. In this study, we have used a threshold value 0 for discrimination of the approved and experimental drugs.

The SVM score is categorized into three groups:

a) Very High: used when the score is >1 (drug-like) and < −1.0 (Non drug-like).

b) High: used when the score is between 0.5-1.0 (drug-like) and in between −1.0 to −0.5 (Non drug-like).

c) Low: when the score lies in between 0–0.5 (drug-like) and in between −0.5 to 0 (Non drug-like).

False positive rate has been calculated via 30 times shuffling the dataset in five fold cross-validation and the average value of FPR is 9.64% (Additional file 2: Table-S2).

Comment-3: How many distinct structural families in drugbank3.0? How structurally diverse of this dataset? Are there many drugs having similar structures? If the answer is yes, will it bias the fingerprint selection and model creation?

Response: We are thankful for this valuable comment. After getting this comment, we analyzed the structural family of drugs in drugbank3.0 and found that at present these were classified into 233 different families (http://www.drugbank.ca/drug_classes). This clearly shows the dataset is highly diverse and suitable for model development.

Comment-4: I tried the example on the web server. But it seems slow and could not give me the result. Is this server really functional?

Response: We are thankful to the reviewer for this comment. Now, the server is completely functional.

Comment-5: Will it possible to have a standalone version of the web server? It will be great if there is a standalone version available to the community.

Response: We are thankful for such a nice suggestion. To improve the visibility of this work, we have developed a standalone version of this software. This is available to the users at http://osddlinux.osdd.net.

Comment-6: On page 1, "can predict drug-likeness of molecules with precession." Is "precession" a typo?

Response: We are thankful to the reviewer for pointing out this typo error. In the revised version, we have corrected this mistake and also take care of any other grammatical error.

Comment-7: I am not sure if this topic is suitable for this computational biology-centric journal. Maybe, this work is more suitable for publishing in journals like BMC.

Response: We are thankful for this suggestion and we think this kind of work is well suited for this journal.

Quality of written English: Acceptable

### Reviewer number 3: Mr Ahmet Bakan (nominated by Prof James Faeder)

Comment-1: The authors developed various classification models using an exhaustive set of chemical fingerprints for discriminating approved drugs from experimental drugs and made these models available via a web server. In the past years, many newly approved drug molecules are breaking the widely accepted rule of 5 for drug-likeness, this improving and updating methods for calculating drug-likeness is an important problem. However, I don't understand why authors developed models that discriminate "approved" drugs from "experimental" drugs. Experimental drugs are molecules that are under investigation. Being experimental does not meet the compound is not drug-like, so any model that discriminates approved from experimental does not have any value. The exhaustive approach would be valuable if models were developed to discriminate drug-like, safe compounds from potentially toxic, non-drug-like compounds.

Response: We completely agreed with the reviewer comment. Although, studies have been done previously with focused towards the discrimination of drug-like molecules from non-drug-like ones. But most of these were based on the use of commercial dataset like MDDR, CMC as drug-like and ACD as non drug-like dataset. Thus, availability of the dataset is the major issue. In contrast, our method is an attempt to discriminate two closely related drug-like molecules. This will be an advance step in drug design process because despite the *in vitro* drug-like properties, many drugs failed in clinical trial (experimental stage). Thus, it is very important to discriminate these two classes of molecules. This is the only dataset that is available for public use and will be an excellent asset for development of public domain servers.

Quality of written English: Not suitable for publication unless extensively edited

Response: We are thankful to the reviewer for this comment. In the revised version, we have tried our best to improve quality of English in revised version of manuscript. Hopefully, the revised version will be suitable for publication.

## Response to the Reviewers’ comments after revision

### Reviewer number 1: Dr Robert Murphy

The authors did not respond adequately to my concern about overfitting. By using the results from cross-validation to make choices (such as which features to use), the expected accuracy of the system so configured is no longer the cross-validation accuracy for that configuration. Simply adding more cross-validation trials does not address the issue. The problem may be clarified by considering that some combination of features and model parameters will optimize performance on any finite dataset but that the same combination may not be optimal for another finite dataset even if chosen from the same underlying distribution. Optimization of these choices does not allow the accuracy to be estimate for the new dataset. The point is that in order for cross-validation to be used to estimate future performance, all choices must be made using the training set only. The observation that the performance on the independent dataset (from DrugBank v3.0) was significantly worse suggests that the two datasets may have been drawn from different distributions (likely) but also that the cross-validation accuracy from the original dataset was an overestimate.

Response: After getting above comments on our revised version, we recheck reviewers comment and our previous response. We realize that we misunderstood comments, this is the reason we make more cross-validation trials. We agree with reviewers that we perform feature selection from whole dataset so there is biasness in feature selection. In this version of manuscript, we also evaluated performance of our models to avoid the ambiguity of biasness. We randomly picked 20% of the data from the whole dataset and called this dataset as validation dataset (for detail see Methods section). Remaining dataset (80% data of whole dataset) called New training dataset, were used for training, testing and evaluation of our models using five-fold cross validation. Now, each and everything such as parameter optimization, feature selection, model building was done on New training dataset (80% dataset). Final model with optimized parameters and features was used to evaluate performance on validation dataset (this dataset never used in any kind of training process or feature selection). The performance of our models on training and validation is shown in Table 6. As shown in our results on validation dataset are in agreement with training dataset. We also observed that the prediction performance of MACCS 159 keys based model is same for the New training and validation dataset as well as model developed on whole training dataset. However, a slight decrease in MCC value from 0.72 to 0.67 on PCA based model and 0.67 to 0.62 on CfsSubsetEval based model was observed for New Training and validation dataset. This implies that model developed on 159 MACCS keys is suitable for further prediction because the prediction accuracy is highly similar on both New Train and validation dataset. These results suggested that the models developed in this study are not over-optimized.

Quality of written English: Acceptable

### Reviewer number 2: Prof Difei Wang (nominated by Dr Yuriy Gusev)

The authors' responses for my questions are acceptable. However, it seems the server still has some problems running examples for virtual screening and design analogs. If possible, it is better to give an estimate of running time. Then the users could decide if they should wait for the results. The output of search database is kind of confusing. The first column gives molecule no. What is this for? Why did the example give the same molecule no. (2) for both ZINC00000053 and CHEMBL505943? It is better to show both database IDs and the corresponding structures.

Response: We are thankful to the reviewer for this valuable suggestion. We have rectified the bug regarding the confusion created by assigning the same molecule number to different compounds. We have also implemented the applet for visualization of chemical structure. Now, the user by clicking on the ID of molecule could visualize the structure of chemical compound. We have also provided the estimated time on the webserver to complete a job. Additionally, an email option has been provided in the webserver. Thus, user will receive a mail after finishing the job.

Quality of written English: Acceptable

## Competing interests

The authors declare that they have no competing interests.

## Authors’ contributions

DS and SKD carried out the data analysis and interpretation, developed computer programs. DS and AM wrote the manuscript. SKD developed the web server and created datasets. GPSR conceived and coordinated the project, guided its conception and design, helped in interpretation of data, refined the drafted manuscript and gave overall supervision to the project. All authors read and approved the final manuscript.

## Supplementary Material

Additional file 1 Results of the MACCS based model using Monte Carlo approach and significant MACCS keys description. Click here for file

Additional file 2 Screening results of the DUD dataset having drug-like properties. Click here for file

## References

[B1] Brown D Future pathways for combinatorial chemistry Molecular diversity 1997 2 4 217 222 9249757 10.1007/BF01715637

[B2] Dolle RE Discovery of enzyme inhibitors through combinatorial chemistry Molecular diversity 1997 2 4 223 236 9249758 10.1007/BF01715638

[B3] Gordon EM Libraries of non-polymeric organic molecules Current opinion in biotechnology 1995 6 6 624 631 8791293 10.1016/0958-1669(95)80103-0

[B4] Smith DA van de Waterbeemd H Pharmacokinetics and metabolism in early drug discovery Current opinion in chemical biology 1999 3 4 373 378 10419843 10.1016/s1367-5931(99)80056-8

[B5] van de Waterbeemd H Gifford E ADMET in silico modelling: towards prediction paradise? Nature reviews Drug discovery 2003 2 3 192 204 10.1038/nrd1032 12612645

[B6] Spiro Z Kovacs IA Csermely P Drug-therapy networks and the prediction of novel drug targets Journal of biology 2008 7 6 20 18710588 10.1186/jbiol81 PMC2776392

[B7] Singh N Sun H Chaudhury S Abdulhameed MD Wallqvist A Tawa G A physicochemical descriptor-based scoring scheme for effective and rapid filtering of kinase-like chemical space Journal of cheminformatics 2012 4 1 4 22316383 10.1186/1758-2946-4-4 PMC3299594

[B8] Lipinski CA Lombardo F Dominy BW Feeney PJ Experimental and computational approaches to estimate solubility and permeability in drug discovery and development settings Advanced drug delivery reviews 2001 46 1–3 3 26 11259830 10.1016/s0169-409x(00)00129-0

[B9] Zhang MQ Wilkinson B Drug discovery beyond the 'rule-of-five' Current opinion in biotechnology 2007 18 6 478 488 18035532 10.1016/j.copbio.2007.10.005

[B10] Ganesan A The impact of natural products upon modern drug discovery Current opinion in chemical biology 2008 12 3 306 317 18423384 10.1016/j.cbpa.2008.03.016

[B11] Bickerton GR Paolini GV Besnard J Muresan S Hopkins AL Quantifying the chemical beauty of drugs Nature chemistry 2012 4 2 90 98 10.1038/nchem.1243 PMC3524573 22270643

[B12] Wang J Krudy G Hou T Zhang W Holland G Xu X Development of reliable aqueous solubility models and their application in druglike analysis Journal of chemical information and modeling 2007 47 4 1395 1404 17569522 10.1021/ci700096r

[B13] Wang J Hou T Drug and drug candidate building block analysis Journal of chemical information and modeling 2010 50 1 55 67 20020714 10.1021/ci900398f

[B14] Vistoli G Pedretti A Testa B Assessing drug-likeness – what are we missing? Drug Discov Today 2008 13 7–8 285 294 18405840 10.1016/j.drudis.2007.11.007

[B15] Ajay A Walters WP Murcko MA Can we learn to distinguish between "drug-like" and "nondrug-like" molecules? Journal of medicinal chemistry 1998 41 18 3314 3324 9719583 10.1021/jm970666c

[B16] Sadowski J Kubinyi H A scoring scheme for discriminating between drugs and nondrugs Journal of medicinal chemistry 1998 41 18 3325 3329 9719584 10.1021/jm9706776

[B17] Gillet VJ Willett P Bradshaw J Identification of biological activity profiles using substructural analysis and genetic algorithms Journal of chemical information and computer sciences 1998 38 2 165 179 9538517 10.1021/ci970431+

[B18] Wagener M van Geerestein VJ Potential drugs and nondrugs: prediction and identification of important structural features Journal of chemical information and computer sciences 2000 40 2 280 292 10761129 10.1021/ci990266t

[B19] Frimurer TM Bywater R Naerum L Lauritsen LN Brunak S Improving the odds in discriminating "drug-like" from "non drug-like" compounds Journal of chemical information and computer sciences 2000 40 6 1315 1324 11128089 10.1021/ci0003810

[B20] Mishra H Singh N Lahiri T Misra K A comparative study on the molecular descriptors for predicting drug-likeness of small molecules Bioinformation 2009 3 9 384 388 19707563 10.6026/97320630003384 PMC2728118

[B21] Knox C Law V Jewison T Liu P Ly S Frolkis A Pon A Banco K Mak C Neveu V DrugBank 3.0: a comprehensive resource for 'omics' research on drugs Nucleic acids research 2011 39 D1035 1041 21059682 10.1093/nar/gkq1126 PMC3013709

[B22] Tang K Zhu R Li Y Cao Z Discrimination of approved drugs from experimental drugs by learning methods BMC bioinformatics 2011 12 157 21569562 10.1186/1471-2105-12-157 PMC3120701

[B23] Brian S Everitt and Torsten Hothorn: A Handbook of Statistical Analyses Using R 2006

[B24] Marvin Applethttp://www.chemaxon.com/download/marvin

[B25] Yap CW PaDEL-descriptor: an open source software to calculate molecular descriptors and fingerprints Journal of computational chemistry 2011 32 7 1466 1474 21425294 10.1002/jcc.21707

[B26] The definitions of MDL’s 166 MACCS search keys can be found from ISIS/Base Help file under “Remote QB in a Molecule Database: Searching Concepts/Examples” at the section 49.2.4: Specifying Searchable Keys as a Query

[B27] Svetnik V Liaw A Tong C Culberson JC Sheridan RP Feuston BP Random forest: a classification and regression tool for compound classification and QSAR modeling Journal of chemical information and computer sciences 2003 43 6 1947 1958 14632445 10.1021/ci034160g

[B28] Garg A Tewari R Raghava GP KiDoQ: using docking based energy scores to develop ligand based model for predicting antibacterials BMC bioinformatics 2010 11 125 20222969 10.1186/1471-2105-11-125 PMC2841597

[B29] Singla D Anurag M Dash D Raghava GP A web server for predicting inhibitors against bacterial target GlmU protein BMC pharmacology 2011 11 5 21733180 10.1186/1471-2210-11-5 PMC3146400

[B30] Hall M Frank E Holmes G Pfahringer BR Willet HI The WEKA data mining software: an update ACM SIGKDD Explorations 2009 11 1 9

