# Supplementary material for: DrugMint: a webserver for predicting and designing of drug-like molecules
Source: Biol Direct. 2013 Nov 5;8:28. doi: 10.1186/1745-6150-8-28 (PMC3826839; doi:10.1186/1745-6150-8-28)
Supplement: Additional file 1 — Results of the MACCS based model using Monte Carlo approach and significant MACCS keys description. [file 1745-6150-8-28-S1.doc]

**Table-S1. The description of the highly desirable MACCS keys**

| **Sr. No.** | **MACCS Key** | **Description** |
| --- | --- | --- |
| 1 | MACCS66 | A tetrahedral carbon atom connected with 3 carbons and one (that may or may not be carbon) atom |
| 2 | MACCS112 | Any atom connected with four atoms by any kind of bond (single, double or triple) |
| 3 | MACCS122 | A nitrogen atom joined with 3 other atoms by any kind of bond |
| 4 | MACCS138 | An aliphatic carbon connected with 3 atoms of which one atom is not the carbon or hydrogen, second is any atom and third is with 2 further hydrogen’s |
| 5 | MACCS144 | Any four atoms connected by non-aromatic bonds |
| 6 | MACCS150 | Any four atoms connected of which atom 1,2 and 3,4 connected by non-ring bond and atom 2,3 joined by ring bond |

**Table-S2**: Results of the 5-fold cross validation by randomly shuffling training dataset 30 times

| **Cycles** | **TP** | **FP** | **TN** | **FN** | **Sen** | **Spec** | **FPR** | **ACC** | **MCC** |
| --- | --- | --- | --- | --- | --- | --- | --- | --- | --- |
| 1 | 1181 | 304 | 2902 | 166 | 87.68 | 90.52 | 9.48 | 89.68 | 0.76 |
| 2 | 1187 | 307 | 2899 | 160 | 88.12 | 90.42 | 9.58 | 89.74 | 0.76 |
| 3 | 1182 | 315 | 2891 | 165 | 87.75 | 90.17 | 9.83 | 89.46 | 0.76 |
| 4 | 1189 | 320 | 2886 | 158 | 88.27 | 90.02 | 9.98 | 89.5 | 0.76 |
| 5 | 1167 | 316 | 2890 | 180 | 86.64 | 90.14 | 9.86 | 89.11 | 0.75 |
| 6 | 1183 | 298 | 2908 | 164 | 87.82 | 90.7 | 9.3 | 89.85 | 0.77 |
| 7 | 1195 | 310 | 2896 | 152 | 88.72 | 90.33 | 9.67 | 89.85 | 0.77 |
| 8 | 1182 | 322 | 2884 | 165 | 87.75 | 89.96 | 10.04 | 89.3 | 0.75 |
| 9 | 1171 | 319 | 2887 | 176 | 86.93 | 90.05 | 9.95 | 89.13 | 0.75 |
| 10 | 1184 | 298 | 2908 | 163 | 87.9 | 90.7 | 9.3 | 89.87 | 0.77 |
| 11 | 1174 | 306 | 2900 | 173 | 87.16 | 90.46 | 9.54 | 89.48 | 0.76 |
| 12 | 1183 | 319 | 2887 | 164 | 87.82 | 90.05 | 9.95 | 89.39 | 0.76 |
| 13 | 1190 | 311 | 2895 | 157 | 88.34 | 90.3 | 9.7 | 89.72 | 0.76 |
| 14 | 1198 | 304 | 2902 | 149 | 88.94 | 90.52 | 9.48 | 90.05 | 0.77 |
| 15 | 1182 | 307 | 2899 | 165 | 87.75 | 90.42 | 9.58 | 89.63 | 0.76 |
| 16 | 1189 | 321 | 2885 | 158 | 88.27 | 89.99 | 10.01 | 89.48 | 0.76 |
| 17 | 1179 | 306 | 2900 | 168 | 87.53 | 90.46 | 9.54 | 89.59 | 0.76 |
| 18 | 1190 | 315 | 2891 | 157 | 88.34 | 90.17 | 9.83 | 89.63 | 0.76 |
| 19 | 1189 | 295 | 2911 | 158 | 88.27 | 90.8 | 9.2 | 90.05 | 0.77 |
| 20 | 1184 | 320 | 2886 | 163 | 87.9 | 90.02 | 9.98 | 89.39 | 0.76 |
| 21 | 1181 | 300 | 2906 | 166 | 87.68 | 90.64 | 9.36 | 89.76 | 0.76 |
| 22 | 1185 | 322 | 2884 | 162 | 87.97 | 89.96 | 10.04 | 89.37 | 0.76 |
| 23 | 1183 | 307 | 2899 | 164 | 87.82 | 90.42 | 9.58 | 89.66 | 0.76 |
| 24 | 1176 | 299 | 2907 | 171 | 87.31 | 90.67 | 9.33 | 89.68 | 0.76 |
| 25 | 1182 | 301 | 2905 | 165 | 87.75 | 90.61 | 9.39 | 89.76 | 0.76 |
| 26 | 1183 | 315 | 2891 | 164 | 87.82 | 90.17 | 9.83 | 89.48 | 0.76 |
| 27 | 1182 | 304 | 2902 | 165 | 87.75 | 90.52 | 9.48 | 89.7 | 0.76 |
| 28 | 1187 | 296 | 2910 | 160 | 88.12 | 90.77 | 9.23 | 89.98 | 0.77 |
| 29 | 1185 | 308 | 2898 | 162 | 87.97 | 90.39 | 9.61 | 89.68 | 0.76 |
| 30 | 1189 | 306 | 2900 | 158 | 88.27 | 90.46 | 9.54 | 89.81 | 0.77 |
| **Average** | **1183.73** | **309.03** | **2896.97** | **163.27** | **87.88** | **90.36** | **9.64** | **89.63** | **0.76** |
| **s.d.** | **6.42** | **8.41** | **8.41** | **6.42** | **0.48** | **0.26** | **0.26** | **0.24** | **0.01** |
